# Dr. Grandy Is Made Surgeon

**Published:** 1898-08

**Authors:** 


### DR. GRANDY IS MADE SURGEON.

Dr. Luther B. Grandy, a most prominent and popular physician of Atlanta, was recently appointed by Governor Atkinson surgeon of the Third Regiment, Georgia Volunteers. Dr. Grandy will rank as major and will have under him two assistants, each of whom will hold the rank of captain.—*Atlanta Constitution.*

During Dr. Grandy's absence Dr. Dunbar Roy will act as editor of the *Atlanta Medical and Surgical Journal.*

